# Frequency of mesiodens in the pediatric population in North India: A radiographic study

**DOI:** 10.4317/jced.51162

**Published:** 2013-12-01

**Authors:** Santosh Patil, Yaspal Pachori, Sumita Kaswan, Suneet Khandelwal, Lalit Likhyani, Sneha Maheshwari

**Affiliations:** 1 Dept of Oral Medicine and Radiology. Jodhpur Dental College, Jodhpur National University, Jodhpur (Raj). India; 2 Dept of Orthodontics. Jodhpur Dental College, Jodhpur National University, Jodhpur (Raj). India; 3 Dept of Conservative Dentistry and Endodontics. Jodhpur Dental College, Jodhpur National University, Jodhpur (Raj). India; 4 Dept of Oral Pathology and Microbiology. Desh Bhagat Dental College, Muktsar. India; 5 Dept of Conservative Dentistry and Endodontics. Jaipur Dental College, Jaipur (Raj). India

## Abstract

Objectives: Mesiodens are the most common supernumerary teeth, occurring in 0.15% to 2.2% of the population. The aim of the present study was to analyze the frequency and radiological features of mesiodens in the pediatric population. Material and methods: The study was based on the radiographic evaluation of 4133 pediatric patients of the age range of 4-15 years, attending the Department of Oral Medicine and Radiology during the time period between September 2008 to December 2012. In addition to the presence of a supernumerary tooth between the 2 central incisors, data regarding the number, position, shape and associated complications were also recorded. Results: The prevalence of mesiodens in the present study was 1.4%. The prevalence was estimated using a 95% confidence interval. The ratio of boys to girls was 1.8:1 and majority of cases (89.7%) had 1 mesiodens. Most of the mesiodens (59.6%) were aligned in a vertical position. 39 mesiodens (62.9%) were impacted, while 14 (22.6%) were partially erupted and only 9 (14.5%) were completely erupted into occlusion. The main complication associated with the mesiodens was midline diastema (28.6%) and 16 patients were asymptomatic. Conclusion: Mesiodens can result in spacing in the arch, delayed or ectopic eruption of the permanent incisors, further altering the occlusion and esthetics of the patient or may remain asymptomatic. It is therefore important for the practitioners to diagnose a mesiodens early in development to allow for optimal treatment plan.

** Key words:**Mesiodens, prevalence, pediatric population, midline diastema.

## Introduction

While supernumerary tooth may be found in any region of the dental arch, the most common site is the midline between the two maxillary central incisors, where it is referred as mesiodens (1). They account for 80% of all supernumerary teeth (2). Mesiodens can occur either singly or multiply (3). A mesiodens may erupt normally, or remain impacted, with a conical crown and a single root, and appear inverted or take a horizontal position (4). On the basis of the morphology, mesiodens can be conical, supplemental and tuberculate type (5). Asymptomatic unerupted mesiodens may be discovered during clinical and radiological examination of the maxillary anterior region by periapical and panoramic radiographs. Maxillary occlusal radiographs also are highly recommended for pediatric patients with dental anomalies in the anterior region of the maxilla (6).

Mesiodens may give rise to a variety of complications, such as impaction of the incisors, delayed and ectopic eruption of adjacent teeth, crowding or midline diastema, axial rotation or displacement or inclination of erupted permanent incisors, resorption of the root of adjacent teeth and development of dentigerous cyst (3,4,6). The objective of the present study was to determine the frequency of mesiodens among a group of pediatric patients in an Indian population along with other features in relation to mesiodens.

## Material and Methods

The study was based on the radiographic evaluation of 4133 pediatric patients who attended the Department of Oral Medicine and Radiology of Jodhpur Dental College General Hospital between September 2008 to December 2012. Ethical committee clearance was obtained from the concerned authority. The parent’s consent was also obtained prior to the inclusion of the subject for the study. The age range of the patients was from 4 to 15 years. Only those patients who visited the out patient department of Jodhpur Dental College General Hospital for treatment of caries, gingival conditions, tooth fracture, malocclusion or routine check up during the period and had no history of any previous extraction or tooth loss due to trauma, were included in the study. Patients with any congenital syndromes were not included in the study.

Radiographic examination was based on the intra oral periapical radiographs and occlusal radiographs of the premaxilla. Radiographs were examined on standard light boxes to determine presence of mesiodens, which was noted as an unerupted supernumerary tooth, or tooth bud between the two central incisors, present in the midline of the maxilla either unilaterally or bilaterally. In addition to the presence of a supernumerary tooth between the two central incisors, data regarding the number, position, shape and associated complications were also recorded.

## Results

Of the 4133 children 57 had mesiodens, of which 37 (65.3%) were boys and 20 (34.7%) were girls. The ratio of boys to girls was 1.8:1 and most of the mesiodens were discovered in the age group of 6-9 years. The prevalence of mesiodens was 1.4%. Most of the cases (89.7%) had 1 mesiodens. Bilateral mesiodens was found in only 5 patients ( Table 1). None of the patients had 3 or more mesiodens. 37 (59.6%) of the mesiodens were aligned in a vertical position, 16 (25.8%) were inverted and 9 (14.6%) were horizontally placed. 39 mesiodens (62.9%) were impacted, while 14 (22.5%) were partially erupted and only 9 (14.6%) were completely erupted into occlusion. Among the 62 mesiodens 48 (77.4%) were conical in shape, which was the most commonly seen followed by supplemental (11 mesiodens, 17.7%) and 3 (4.9%) were tuberculate in shape. The main complication associated with the mesiodens was midline diastema seen in 14 patients (28.6%) followed by delayed eruption of permanent central incisors (13 patients, 22.5%), axial rotation or inclination of permanent central incisors (9 patients, 16.3%) and resorption of adjacent teeth (5 patients, 8.1%). 16 patients were asymptomatic.

**Table 1:**
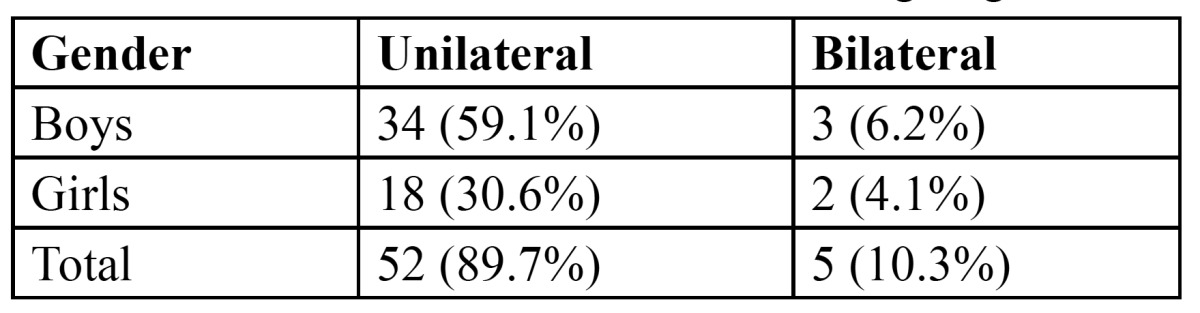
Distribution of mesiodens according to gender.

## Discussion

Supernumerary teeth are developmental anomalies of number which are observed during regular dental checkup. It affects both dentitions equally, but the permanent dentition has a higher incidence of the anomaly. Mesiodens is the most frequently observed dental anomaly in the permanent dentition (2). The prevalence of mesiodens has been approximated to be 0.15% to 2.2% in the literature (7). There have been various studies involving different populations, which have been published till date. According to these studies the prevalence of mesiodens is 0.45% in Caucasians (7), 1.43% in Norwegians (8), 0.3% in Turkish population (9) and 0.4% in Finnish population (10). The results of the present study are in line with other findings as reported in the literature. The prevalence was 1.4% in the present study. Although, much higher prevalence has also been reported in a Turkish population in a study by Ersin et al. (11). They reported a prevalence of 8.3% in the permanent dentition, 4.2% in the primary dentition and 87.5% in the mixed dentition.

Mesiodens occurs more in boys as compared to girls, as is seen in the present study. Out of the 57 cases with mesiodens, 37 were boys and only 20 girls. The ratio of boys to girls was 1.8:1. Most studies have reported that the sex ratio was 2:1, which is similar to the present study (2,4,12,13). While, Huang et al. (14), and Ersin et al. (11), showed a higher sex distribution ratio. Supernumerary teeth in primary dentition are noted with an almost even gender distribution. The differences in the gender distribution may be due to the different sample sizes and racial group that were examined. Majority of the cases in the present study were of the age 6-9 years at the time of examination. This coincides with the age at which mesiodens show maximum prevalence. This time period coincides with the eruption time of the maxillary central incisors. It was thus obvious that most cases were discovered during this period. Radiographic evaluation was done as an aid to rule out any congenitally missing teeth, supernumerary teeth, any associated pathology such as cysts or tumors, when there was delaying of the eruption of the permanent maxillary central incisors or observance of any malpositioning of teeth.

In the present study, there were unilateral or single mesiodens in majority of cases (89.7%). There were only 5 cases that had 2 mesiodens. Not a single case with more than 2 mesiodens was observed during the study. These findings are in conjunction with the findings of Asaumi et al. (15), Gunduz et al. (7), Kim et al. (6), and Huang et al. (14), who reported one mesiodens in most of the reported cases. Most of these mesiodens remain impacted and are discovered accidentally during the radiographic examination. However about 25% have shown to erupt (7,8). 62.9% of the total mesiodens were impacted and 37% either erupted fully or partially in the present study, which was in line with other studies mentioned in the literature.

The shape of the mesiodens may vary from being a simple conical shape to a more complicated crown with tubercles. Conical mesiodens is mostly peg shaped, with root formation ahead of or at an equivalent stage to that of the central incisor. Conical form is more common and it mostly erupts as a diminutive but completely formed tooth. In the present study also, conical shape was most commonly observed (77.4%), which corresponds with the findings in the literature (3,4,6). Tuberculate mesiodens is also common in permanent dentition and usually do not erupt. They develop later and show delayed or incomplete root formation when compared with the adjacent teeth. These multicusped mesiodens often interferes with the eruption of incisors. A supplemental mesiodens is more commonly seen in primary dentition. It resembles the tooth of the normal series and is rarely unerupted (5,14-16).

With regard to the direction of growth, Liu (12), Gunduz et al. (7), Roychoudhary et al. (17), and Asaumi et al. (15), found that 46%, 55.2%, 62.5% and 67% of the supernumerary teeth were in a vertical position axis. In the present study, 59.6% of all mesiodens were oriented in a vertical direction, 25.8% in an inverted position and 14.6% in a horizontal direction. Thus, the present study also showed higher frequencies of vertically aligned mesiodens as was observed with other similar studies.

Mesiodens is capable of causing a variety of clinical complications which include interference with the eruption and positioning of the adjacent teeth, delayed or non eruption of maxillary incisors, midline diastema, axial rotation or inclination of erupted permanent maxillary incisors, root resorption of adjacent teeth, dentigerous cyst formation, intraoral infection and mesiodens pulpitis (6,7). In the present study, complications observed were midline diastema (28.6%) followed by delayed eruption of permanent central incisors (22.5%), axial rotation or inclination of permanent central incisors (16.3%) and resorption of adjacent teeth (8.1%). 16 patients were asymptomatic. Dentigerous cyst, root anomaly, mesiodens pulpitis and intraoral infection were not observed in the present study. In contrast to this, various other studies have shown evidence of these complications. This is probably due to the fact that these complications occur in long-standing cases. The present study involved only the pediatric population. Asaumi et al. (15), reported 19 cases of dentigerous cysts out of the 51 cases of mesiodens, aged above 20 years and only 3 in patients below 20 years of age. Similar findings have been reported by Primosch (5), who showed follicular enlargement in 30% cases. Various authors have reported that mesiodens can delay or prevent eruption of central incisors in 26-52% of the cases and displacement or rotation of the adjacent teeth in 28-63% of the cases (3,5). These results support the findings of the present study.

A thorough clinical and radiological examination aids in the management of mesiodens. However, very often there can be confusion about whether and when to surgically remove the mesiodens, or whether they should be left in position and radiographic follow-up should be done at regular time intervals. Whatsoever be the management approach, early diagnosis is critical. Russell and others recommended extraction of mesiodens in the early mixed dentition stage for better positioning of teeth and subsequently reducing the need for orthodontic treatment (3-5,17). Some believe that the appropriate time for removal of mesiodens is 8-9 years when the maxillary incisors erupt. Removal at this age is advantageous and easy for the clinician as the behavior of a child is much easier to manage and the anesthesia required can be less invasive. Delayed extraction of mesiodens can also be done when the root formation of the adjacent permanent incisors have completed. Surgical removal should be avoided if the mesiodens remains asymptomatic or when there is an increased risk of damaging the developing permanent incisors. However, a periodic follow-up is necessary (18).

## Conclusion

The prevalence of mesiodens in the pediatric population was 1.4%. it occurs more frequently in boys, the ratio being 1.8:1 in the present study. The majority of the mesiodens were conical in shape and had a vertical direction of growth. 62.9% of the cases were unerupted. Only 5 children (9.3%) had 2 mesiodens bilateral to the midline. The role of the clinician in the successful management of a mesiodens is important because the earlier the diagnosis, minimal the future complications and the better is the prognosis.

## References

[1] Sykaras SN (1975). Mesiodens in primary and permanent dentitions. Report of a case. Oral Surg Oral Med Oral Pathol.

[2] Ferrĕs-Padrŏ E, Prats-Armengol J, Ferrĕs-Amat E (2009). A descriptive study of 113 unerupted supernumerary teeth in 79 pediatric patients in Barcelona. Med Oral Patol Oral Cir Bucal.

[3] Russell KA, Folwarczna MA (2003). Mesiodens- diagnosis and management of a common supernumerary tooth. J Can Dent Assoc.

[4] Zhu JF, Marcushamer M, King DL, Henry RJ (1996). Supernumerary and congenitally absent teeth: A literature review. J Clin Pediatr Dent.

[5] Primosch RE (1981). Anterior supernumerary teeth: assessment and surgical intervention in children. Pediatr Dent.

[6] Kim SG, Lee SH (2003). Mesiodens: A clinical and radiographic study. ASDC J Dent child.

[7] Gündüz K, Celenk P, Zengin Z, Sümer P (2008). Mesiodens: A radiographic study in children. J Oral Sci.

[8] Humerfelt D, Hurlen B, Humerfelt S (1985). Characteristics of premaxillary hyperdontia. A radiographic study. Acta Odontol Scand.

[9] Buvenviaje TM, Rapp R (1984). Dental anomalies in children: a clinical and radiographic survey. ASDC J Dent Child.

[10] Jarvinen S, Lehtinen L (1981). Supernumerary and congenitally missing primary teeth in Finnish children. An epidemiologic study. Acta Odontol Scand.

[11] Ersin NK, Candan U, Alpoz AR, Akay C (2004). Mesiodens in primary, mixed and permanent dentitions: a clinical and radiographic study. J Clin Pediatr Dent.

[12] Liu JF (1995). Characteristics of premaxillary supernumerary teeth: A survey of 112 cases. ASDC J Dent Child.

[13] Fenández-Montenegro P, Valmaseda-Castellón E, Beruni Aytés L, Gay Escoda C (2006). Retrospective study of 145 supernumerary teeth. Med Oral Patol Oral Cir Bucal.

[14] Huang WH, T Sai TP, Su HL (1992). Mesiodens in the primary dentition stage: A radiographic study. ASDC J Dent Child.

[15] Asaumi JI, Shibata Y, Yanagi Y, Hisatomi M, Matsuzaki H, Konouchi H (2004). Radiographic examination of mesiodens and their associated complications. Dentomaxillofac Radiol.

[16] Mukhopadhyay S (2011). Mesiodens: A clinical and radiographic study in children. J Indian Soc Pedod Prev Dent.

[17] Roychoudhary A, Gupta Y, Prakash H (2000). Mesiodens: a retrospective study of fifth teeth. J Indian Soc Pedod Prev Dent.

[18] Mitchell L, Bennett TG (1992). Supernumerary teeth causing delayed eruption- a retrospective study. Br J Orthod.

